# The influence of past experiences on future willingness to perform bystander cardiopulmonary resuscitation

**DOI:** 10.1186/s12245-019-0256-5

**Published:** 2019-12-12

**Authors:** Keng Sheng Chew, Shazrina Ahmad Razali, Shirly Siew Ling Wong, Aisyah Azizul, Nurul Faizah Ismail, Sharoon Juliet Kun Chyee Ak Robert, Yegharaj A/L Jayaveeran

**Affiliations:** 1 Faculty of Medicine of Health Sciences, Universiti Malaysia Sarawak, 94300 Kota Samarahan, Sarawak Malaysia; 2 Faculty of Economics and Business, Universiti Malaysia Sarawak, 94300 Kota Samarahan, Sarawak Malaysia

**Keywords:** Bystander cardiopulmonary resuscitation, Pay-it-forward, Cardiac arrest

## Abstract

**Background:**

The influence of past familial experiences of receiving cardiopulmonary resuscitation (CPR) and medical help in various cardiac arrest and nonfatal cardiac events toward willingness to “pay it forward” by helping the next cardiac arrest victim was explored.

**Methods:**

Using a validated questionnaire, 6248 participants were asked to rate their willingness to perform bystander chest compression with mouth-to-mouth ventilation and chest compression-only CPR. Their past familial experiences of receiving cardiopulmonary resuscitation (CPR) and medical help in various cardiac arrest and nonfatal cardiac events were also recorded.

**Results:**

Kruskal-Wallis test with post hoc Dunn’s pairwise comparisons showed that the following were significantly more willing to perform CPR with mouth-to-mouth ventilation: familial experience of “nonfatal cardiac events” (mean rank = 447) vs “out-of-hospital cardiac arrest with no CPR” (mean rank = 177), U = 35442.5, z = −2.055, *p* = 0.04; “in-hospital cardiac arrest and successful CPR” (mean rank = 2955.79) vs “none of these experiences” (mean rank = 2468.38), U = 111903, z = −2.60, *p* = 0.01; and “in-hospital cardiac arrest with successful CPR” (mean rank = 133.45) vs “out-of-hospital arrest with no CPR” (mean rank = 112.36), U = 4135.5, z = −2.06, *p* = 0.04. For compression-only CPR, Kruskal-Wallis test with multiple runs of Mann-Whitney U tests showed that “nonfatal cardiac events” group was statistically higher than the group with “none of these experiences” (mean rank = 3061.43 vs 2859.91), U = 1194658, z = −2.588, *p* = 0.01. The groups of “in-hospital cardiac arrest with successful CPR” and “in-hospital cardiac arrest with transient return of spontaneous circulation” were the most willing groups to perform compression-only CPR.

**Conclusion:**

Prior familial experiences of receiving CPR and medical help, particularly among those with successful outcomes in a hospital setting, seem to increase the willingness to perform bystander CPR.

## Introduction

As 80% of out-of-hospital cardiac arrest (OHCA) cases happened at home [1] as a result of cardiovascular diseases [2], it is imperative to educate the general public on the skills of bystander cardiopulmonary resuscitation (CPR) [3]. Studies have shown that bystander CPR improves the chance of survival of OHCA by up to two to three times [4]. Despite that, the rate of bystander CPR has not been as encouraging as we would like it to be [5–7].

A number of studies had been conducted to identify factors influencing the willingness of bystanders to perform CPR [6–14]. These factors can generally be divided into two broad categories, i.e., (1) bystander factors and the (2) victim factors. Bystander factors that increase the willingness to perform bystander CPR include bystanders’ prior CPR training [6, 7, 10], bystander’s educational and income level [10–12], and the bystanders’ emotional state at the time of the incident [10, 13]. Victim factors that increase the chance of having bystander CPR performed include the familiarity and the relationship of the victim to the bystander [8, 9, 14]: a child victim [8] and the perceived cleanliness of the victim [9]. One factor that is conspicuously less explored, however, is the influence of the bystanders’ past familial experiences of receiving CPR and medical help in various IHCA or OHCA conditions and nonfatal cardiac events on his or her future willingness to perform bystander CPR.

“Pay-it-forward” is an expression in which a recipient of an act of kindness reciprocates by repaying this kind act to someone else rather than to the original benefactor [15]. This concept was crisply captured by author Lily Hardy Hammond as early as 1916, when she famously wrote in her book, *In the Garden of Delight* [16], “You don't pay love back; you pay it forward.” Undergirding the motivation to pay it forward is the feeling of indebtedness for the help that one has received [17]. In the context of disasters, Atsumi (2014) and Daimon (2018) demonstrated that survivors of the Great East Japan Earthquake in 2011 who had received help were more likely to render help by volunteering to help out in future disasters [17, 18]. According to Atsumi (2014), these survivors seemed to be relieved by volunteering to assist in other disaster events [17].

We are not certain if help received in a prior cardiac arrest event may play an important role in influencing bystanders to perform CPR (similar to the “pay-it-forward” mechanism discussed above). Hence, we embarked on this study with the primary objective of exploring whether prior familial experience with cardiac arrests (with or without bystander CPR) or nonfatal cardiac events has a significant effect in increasing the willingness to perform bystander CPR. The secondary objectives of this study are to explore the influence of four personal characteristics (i.e., gender, involvement in a medical nongovernmental organizations (NGOs) such as Red Crescent Malaysia, St. John Ambulance Malaysia, Civil Defence, etc., their prior CPR training, and their prior experience of administering bystander CPR toward their willingness to perform bystander CPR.

## Materials and methods

### Participants

The respondents of this survey were the adult participants of a mass CPR event held on 20 September 2017 in Universiti Malaysia Sarawak (UNIMAS), which is a public university located in the state of Sarawak, Malaysia [19]. Convenience sampling was applied. Prior informed consent was obtained from the participants before starting this survey. The participants were assured that no personal data such as their names and national identification numbers or passport numbers would be collected. Approval to conduct this study was obtained from institutional research ethics board of UNIMAS (reference no: UNIMAS/NC-21.02/03-02 Jld.3 [94]).

### Materials

A self-administered questionnaire comprises of three parts, i.e., the demographic data (Part A), public knowledge of CPR (Part B), and general public attitudes toward CPR (Part C) was used in this survey. The draft of the questionnaire was first constructed by a panel of experts consisting of emergency physicians and trainers of basic life support. Validation of the questionnaire was then performed by ten clinical lecturers from Faculty of Medicine and Health Science, UNIMAS, in order to determine its internal consistency as well as its inter-rater reliability. The Cronbach alpha of this questionnaire is 0.93 indicating good internal consistency of the items. The intra-class correlation coefficient is 0.93, indicating good inter-rater reliability.

With regard to past familial (or even personal) experiences with nonfatal cardiac events and cardiac arrest, the participants were asked “Have you or any of your family member/loved ones had the following incident before? (1) “Nonfatal heart attack,” i.e., heart attack before but not collapsed; (2) “OHCA with successful CPR,” i.e., collapsed at home and had successful bystander CPR done outside of hospital and subsequently admitted to hospital; (3) “OHCA with unsuccessful CPR,” i.e., collapsed at home, had bystander CPR done but unsuccessful; (4) “OHCA with no CPR,” i.e., collapsed at home with no CPR done; (5) “in-hospital cardiac arrest or IHCA with transient ROSC,” i.e., collapsed in hospital, had CPR done with transient return of spontaneous circulation or ROSC but subsequently passed away in hospital; (6) “had successful CPR done in hospital and subsequently discharged alive” (IHCA with successful CPR); (7) “had unsuccessful CPR done in hospital” (IHCA with unsuccessful CPR); and (8) “none of these experiences or not applicable.” The willingness to perform both chest compression with mouth-to-mouth ventilation (CC + MTM) as well as chest compression-only (CC only) CPR was captured on a Likert scale from “1” (least willing) to 10 (“most willing”).

### Procedure

As mentioned, this anonymous, volitional survey was conducted in conjunction with the 1-day mass CPR educational program which was held from 8:00 am to 8:00 pm (ten sessions) on 20 September 2017 in UNIMAS [19]. While waiting for their practice sessions, the questionnaire forms were distributed to the participants. The authors of this paper and their research assistants were present on that day to respond to any specific query from the participants with regard to the questionnaire.

## Results

A total of 6248 participants participated in the survey. Out of these, 4366 participants (69.8%) were female, and 1871 (29.9%) participants were male (11 participants did not reveal their gender). In terms of their age groups, majority of the participants (3187 or 51%) were below the age of 20 years old, followed by those between 21 to 30 years old (2336 or 37.4%), and 469 participants (7.5%) were between 31 to 40 years old. Only 11 participants (0.2%) were above 60 years old (21 missing data). (See Table 1 for the details of the demographic data of the participants)

**Table 1:** Descriptive data of participants

Variables		Frequency (*n* = 6248)	%
Gender			
	Male	1871	29.9
	Female	4366	69.9
	Missing data	11	0.2
Age group			
	below 20 years old	3187	51.0
	21–30 years old	2336	37.4
	31–40 years old	469	7.5
	41–50 years old	157	2.5
	51–60 years old	67	1.1
	above 60 years old	11	0.2
	Missing data	21	0.3
Occupation			
	Students	4770	76.3
	Professional/technical works	402	6.4
	Administrative and clerical work	138	2.2
	Armed forces	15	0.2
	Elementary occupations	5	0.1
	Plant and machine operators	4	0.1
	Service and sales sector	62	1.0
	Skilled agricultural	37	0.6
	Self-employed	38	0.6
	Retired	15	0.2
	Unemployed	165	2.6
	Missing data	597	9.7
Highest education level			
	No formal education	12	0.2
	Primary	91	1.5
	Secondary	1824	29.2
	Post-secondary (diploma, certificate, etc.)	1794	28.7
	Tertiary	2527	40.4
Member of any medical nongovernmental organizations (NGO)?			
	Yes	697	11.2
	No	5420	86.7
	Missing data	131	2.1

Nonparametric tests were applied in this survey as the normality of the distribution of all data could not be assumed, with Shapiro-Wilk test *p* < 0.05, skewness z value of −2.90 (standard error or SE = 0.05), and kurtosis z value of −4.90 (SE = 0.12) for male participants on their willingness to perform CC only, and for female participants, the skewness and kurtosis z values are −0.45 (SE = 0.04) and −5.31 (SE = 0.08), respectively. Similarly, for willingness to perform CC + MTM, skewness and kurtosis z values are −12.5 (SE = 0.06) and −3.05 (SE = 0.11), respectively, for male participants; and −18.03 (SE = 0.04) and −2.95 (0.07), respectively, for female participants and Shapiro-Wilk test *p* < 0.05.

Overall, the willingness to perform CC only was shown to be higher with median score of 7.00 (inter-quartile range, IQR 5.00, 9.00) as compared to the willingness to perform CC + MTM with the median score of 5.00 (IQR 5.00, 8.00). Mann-Whitney U tests were performed to analyze the association between willingness to perform both CC + MTM and CC only with four personal characteristics of the participants, i.e., (1) their gender, (2) involvement in a medical nongovernmental organizations (NGOs) such as Red Crescent Malaysia, St. John Ambulance Malaysia, Civil Defence, etc., (3) their prior CPR training, and (4) their prior experience of administering bystander CPR toward their willingness to perform bystander CPR. Generally, male gender, participants who were members of medical NGOs, participants who had prior CPR training, and participants who had previous experience of administering bystander CPR reported to be significantly more willing to administer both types of bystander CPR. The details of these results are tabulated in Tables 2 and 3.

**Table 2:** Association of independent variables with the willingness to perform bystander CC + MTM (*n* = 6248)

	Median (Inter-quartile range 25th percentile, 75th percentile)	Mean rank	U-statistics	*p* value
Male	6.00 (5.00, 8.00)	3177.82	2999505	*p* < 0.001
Female	5.00 (5.00, 7.00)	2775.99		
Member of a medical uniformed NGO such as St. John’s ambulance, red crescent, etc.	6.00 (5.00, 8.00)	3029.54	1517177.5	*p* = 0.002
Not a member of any medical uniformed NGO	5.00 (5.00, 8.00)	2817.13		
With previous experience of administering CPR	6.00 (5.00, 8.00)	2877.42	1205596	*p* < 0.001
Without previous experience of administering CPR	5.00 (5.00, 7.00)	2610.13		
With CPR training	6.00 (5.00, 8.00)	2974.04	4039339	*p* < 0.001
Without CPR training	5.00 (5.00, 7.00)	2831.39		

Note: all inferential statistics were performed using Mann-Whitney U test

**Table 3:** Association of independent variables with the willingness to perform bystander CC only (*n* = 6248)

	Median (Inter-quartile range 25th percentile, 75th percentile)	Mean rank	U-statistics	*p* value
Male	7.00 (5.00, 9.00)	3191.08	3949536	*p* = 0.04
Female	7.00 (7.00, 8.00)	3088.11		
Member of a medical uniformed NGO such as St. John’s ambulance, red crescent, etc.	7.00 (5.00, 9.00)	3195.03	1794058.5	*p* = 0.03
Not a member of any medical uniformed NGO	7.00 (7.00, 9.00)	3041.51		
With previous experience of administering CPR	8.00 (5.00, 8.00)	3056.35	13368.22	*p* < 0.001
Without previous experience of administering CPR	7.00 (5.00, 9.00)	2729.54		
With CPR training	7.00 (5.00, 9.00)	3174.20	4039339	*p* < 0.001
Without CPR training	7.00 (5.00, 8.25)	2968.37		

Note: all inferential statistics were performed using Mann-Whitney U test

With regard to the influence of past family experiences with cardiac arrest and nonfatal cardiac events on the willingness to perform CC + MTM, Kruskal-Wallis test showed significant differences (*p* < 0.001) between the mean ranks of at least one pair of the groups, with H(7) = 30.21. Post hoc Dunn’s pairwise comparisons were carried out for the 28 pairs of groups. Significant difference (*p* < 0.001), adjusted using the Bonferroni correction, was found between the group with nonfatal cardiac events versus the group who had “none of these experiences” (*p* < 0.001). Multiple runs of Mann-Whitney U tests subsequently performed for further pairwise comparisons were consistent with this finding. In particular, the score for the group of nonfatal cardiac events (mean rank = 447) was statistically higher than for the group of OHCA with no CPR (mean rank = 177), U = 35442.5, z = −2.055, *p* = 0.04. The group of IHCA with successful CPR (mean rank = 2955.79) was statistically higher than the group with “none of these experiences” (mean rank = 2468.38), U = 111903, z = −2.60, *p* = 0.01. Similarly, the group of IHCA with successful CPR (mean rank = 133.45) was also statistically higher than the group OHCA with no CPR (mean rank = 112.36), U = 4135.5, z = −2.06, *p* = 0.04.

With regard to the influence of past family experiences with cardiac arrest and nonfatal cardiac events on the willingness to perform CC only, Kruskal-Wallis test showed significant differences (*p* = 0.001) between the mean ranks of at least one pair of the groups, with H(7) = 25.34. However, the post hoc Dunn’s pairwise comparisons adjusted using Bonferroni correction on the 28 pairs of groups were not able to detect which pair(s) of groups has or have significant differences. Multiple runs of Mann-Whitney U tests subsequently performed for further pairwise comparisons found that the scores for the group of nonfatal cardiac events was statistically higher than the group with “none of these experiences” (mean rank = 3061.43 vs 2859.91), U = 1194658, z = −2.588, *p* = 0.01. The group of IHCA with successful CPR, however, appears to be most willing to perform CC only as the score for this group was significantly higher when compared to the following four groups: (1) with the group of nonfatal cardiac events (mean rank = 313.91 vs 269.18), U = 12041.5, z = −2.076, *p* = 0.038; (2) with the group of OHCA with unsuccessful CPR (mean rank = 59.21 vs 42.33), U = 872.5, z = −2.878, *p* = 0.004; (3) with the group of OHCA with successful CPR (mean rank = 60.18 vs 47.66), U = 1110.5, z = −2.101, *p* = 0.036; (4) with the group of OHCA with no CPR (mean rank = 146.78 vs 118.93), U = 4379, z = −2.619, *p* = 0.009; and (5) with the group with “none of these experiences” (mean rank = 3277.49 vs 2655.61), U = 119033, z = −3.119, *p* = 0.002.

Similarly, the score for the group of IHCA with CPR and transient ROSC was also significantly higher than that for the following groups: (1) with the group of OHCA with unsuccessful CPR (mean rank = 71.00 vs 43.41), U = 1360.0, z = −2.564, *p* = 0.01; (2) with the group of OHCA with no CPR (mean rank = 154.18 vs 131.52), U = 6784.5, z = −2.204, *p* = 0.03; and (3) with the group with “none of these experiences” (mean rank = 3140.19 vs 2668.00), U = 184264.0, z = −2.822, *p* = 0.005.

With regard to the influence of the participants’ educational level on their willingness to perform CC + MTM, Kruskal-Wallis test showed significant differences (*p* = 0.028) between the mean ranks of at least one pair of the groups, with H(4) = 10.855. Post hoc Dunn’s pairwise comparisons were carried out for the ten pairs of groups. Significant difference (*p* = 0.031), adjusted using the Bonferroni correction, found that participants with tertiary education (mean rank 2983.78) were significantly more willing than participants with post-secondary education (mean rank 2828.56), U = 155.22, z = −2.96, *p* = 0.031. Similarly, with regard to the influence of the participants’ educational level on their willingness to perform CC only, Kruskal-Wallis test showed significant differences (*p* < 0.001) between the mean ranks of at least one pair of the groups, with H(4) = 32.587. Post hoc Dunn’s pairwise comparisons were carried out for the ten pairs of groups. Significant difference (*p* = 0.031), adjusted using the Bonferroni correction, was found between the group with primary education (mean rank = 2447.47) vs post-secondary education (mean rank = 3173.15) (*p* = 0.002); between primary education (mean rank = 2447.47) vs tertiary education (mean rank = 3216.40) (*p* = 0.001); between secondary education (mean rank = 2985.43) vs post-secondary education (mean rank = 3173.15) (*p* = 0.016); and between secondary education (mean rank = 2985.43) and tertiary education (mean rank = 3216.40) (*p* < 0.001).

## Discussion

Our results show that participants with past familial experiences of IHCA with successful CPR or IHCA with CPR and transient ROSC as well as nonfatal cardiac events (did not require CPR) were significantly more willing to perform bystander CPR (both CC + MTM and CC only).

This significant increase in willingness to perform bystander CPR could probably be explained from psychological and sociological points of view. In their arousal: cost-reward model, Dovidio et al. (1991) conceptualized the idea that when one becomes aware that a victim is suffering in an emergency situation, this arouses an emotionally unpleasant experience, and the way to relieve this unpleasant emotion is by rendering help to the victim. This also explains the importance of creating awareness among bystanders that cardiac arrest is an emergency situation that requires prompt intervention [20].

Piliavin and Charng (1990) also show that people are more willing to help after a disaster has occurred to them [21]. This finding is consistent with Blau’s *homo economicus* theory of social exchange [22]. According to Blau (1960), a person who has received help from others often feels obligated to reciprocate. In this regard, “paying it forward” (by helping another cardiac arrest victim) is postulated as a mechanism to relieve such obligation. Similarly, according to the “warm-glow giving” theory by Andreoni (1990), people volunteer because of the sense of joy and personal satisfaction for having helped someone [23]. In this regard, participants who had been helped by others before are more willing to volunteer an altruistic act such as bystander CPR for the next victim for the sense of warm glow that they can derive [23].

Surprisingly, however, familial experiences of OHCA with CPR (even among those with the successful outcome of having the victim revived and subsequently brought to the hospital for further management) did not seem to significantly increase the willingness to perform bystander CPR. The reason for this could not be ascertained, but it could probably be due to the fact that as all victims who had been successfully revived (i.e., achieved ROCS) in an out-of-hospital setting were subsequently brought in to the hospital. Hence, the participants might have attributed to the success (or failure) of the eventual outcomes of resuscitation to the efforts made by hospital staff rather than that of the bystanders.

In this study, we found that male gender as well as educational level seems to have a significant influence on the willingness to perform bystander CPR. Those who have higher educational levels seem to be more willing than those with lower educational level to perform bystander CPR. These findings are consistent with a number of previous studies on volunteerism [24, 25]; although a recent literature review by Haski-Leventhal (2009) found that except for educational level which has been consistently found to be related to increased willingness to volunteer, studies on other sociodemographic factors such as income and gender had led to mixed results [26]. In this study, those who have been trained in CPR or had prior experience as a member of a medical NGO or had administered bystander CPR were also significantly more willing to perform bystander CPR. As demonstrated in a previous study by Shotland and Heinold (1985), logically those who had been trained would be more competent and, hence, more willing to help [27]. Having such a competent bystander around is also important in the sense that his or her mere presence has been demonstrated in previous studies to encourage and empower other less competent bystanders to offer some form help [28–31].

There are a number of pertinent limitations in this study. First, as OHCA victims who had been successfully resuscitated through bystander CPR would eventually be transferred to the hospitals, the participants might have attributed the success (or failure) of CPR based on the eventual outcomes from the hospital particularly if the victim collapsed again in the hospital. Hence, it could be that the eventual outcomes from the hospitals that might influence the willingness of them to render bystander CPR rather than the initial outcomes from bystander CPR in an out-of-hospital setting. Perhaps, a future study could be conducted with more specific subcategories, such as asking the participants on their willingness to perform bystander CPR based on the successful outcome of bystander CPR that their family members received in the out-of-hospital setting per se (regardless of the eventual outcome in the hospital). Secondly, the competency and knowledge of the participants with regard to the prompt need of bystander CPR was not ascertained. This could have affected their willingness to perform bystander CPR even if they had family members whom previously had received bystander CPR. As this study was conducted in a university setting and as demonstrated in the demographic data of the participants, majority of the participants were university students from the younger age brackets. These would have skewed the responses obtained, and hence, the results might not be generalizable to another population. Finally, although a link has been drawn between the results of this study with the “paying-it-forward” mechanism as the possible explanation, this remains a postulation, at best. Perhaps, future study could be conducted to delve into the various reasons on why a bystander would or would not perform bystander CPR, including the possible psychological reasons such as the “paying-it-forward” mechanism discussed above.

## Conclusion

This study suggests that prior familial experiences of receiving CPR, particularly among those with successful outcomes in a hospital setting, seem to increase the willingness to “pay it forward” by volunteering to perform bystander CPR on the next victim. Male participants as well as those who have been trained or administered bystander CPR before or who are members of medical NGOs also seem to be more willing to perform bystander CPR.

## Data Availability

The dataset is not available publicly; however, it may be requested from the corresponding author upon reasonable request.
